# A Multi-Point View of Genetic Factors Affecting Hereditary Transmissibility of Asthma

**DOI:** 10.7759/cureus.28768

**Published:** 2022-09-04

**Authors:** Aryan Kulkarni, Dr. Anupkumar Kediya

**Affiliations:** 1 Pathology, Jawaharlal Nehru Medical College, Datta Meghe Institute of Medical Sciences, Wardha, IND

**Keywords:** transcription, dna methylation, multi-ome, hereditary diseases, epigenomics, gwas, human genetics and epigenetics, genomics, genetic biomarkers, asthma

## Abstract

Asthma is a respiratory illness characterized by episodes of bronchial spasm that make breathing difficult. It often has an association with an allergic response or any hypersensitivity followed by inflammation and hypersensitivity of the airways. Asthma is a sophisticated, complicated, and diverse respiratory condition. Even while heredity is a chief hazard factor for developing asthma, environmental and inner exposures, as well as how they interact with genetic variables, also have a substantial impact on the disease's pathogenesis. In recent years, the field of asthma research has seen the emergence and deployment of high throughput omics techniques for the unbiased screening of biomarkers such as genes, transcripts, proteins, and metabolites. Better asthma risk profile has been achieved by utilizing large-scale studies that are illustrative of various population-based data and merging with clinical data. This allergic airway illness has a wide range of phenotypes and endotypes, many of which have not yet been fully defined. The diversity of phenotypes is reflected in similarly complicated etiologies, and it is thought that a number of genetic and environmental variables interrelate to promote the danger of asthma in both children and adults. In order to achieve this objective, significant efforts are necessary to describe different phenotypes, uncover fundamental mechanisms, and find associated biomarkers. It is clear that the analysis, monitoring, and treatment of asthma require a tailored strategy. The risk of developing a multifactorial ailment is characterized by the grade of genetic relationship between the individual and affected relative. Additionally, the risk is typically larger if the relative has a severe illness or was afflicted when they were young. The asthma phenotype is highly varied and expressed non-linearly in contrast to single gene illnesses. This makes it challenging to predict whether someone with a certain gene or set of genotypes would have asthma.

## Introduction and background

Children and adults around the world are affected by asthma, which is a complicated, heterogeneous disease with a wide range of symptoms and partially known underlying pathogenetic pathways. Many genome-wide association studies (GWASs) over the previous decade have discovered numerous genetic variations linked to asthma risk. These primarily non-coding variations control gene expression and the heredity of asthma. These findings, however, only partially support the relevance of genetics in the development of asthma. It has taken a lot of work to subtype asthma [1]. Observable traits are grouped into categories called phenotypes, which are often the end product of genetics and environment. Asthma phenotypes have long been differentiated based on the seriousness of the condition, its reaction to therapy, and aggravating variables. Phenotypes may not always represent the same molecular and cellular processes that underlie illness [2]. On the contrary, endotypes are disease subgroups based on unique pathophysiological processes. Endotypes may be helpful in the therapeutic setting because they organize the mechanistic knowledge of diverse illnesses such as asthma, guiding therapy toward modalities that target certain pathways that may be damaged within a given endotype [3]. This is crucial for persons with asthma since different drugs work differently for them. In order to properly prescribe medication and enhance asthma management, it is becoming more and more crucial to precisely classify asthma endotypes given the rising accessibility of biologics that target specific pathways. Researchers have recently become interested in the innovative field of epigenetics, which may help us comprehend the disease's causes through epigenetic techniques such as DNA methylation, microRNA expressions, and histone alterations [4]. Complex diseases such as asthma are characterized by a number of cascade of events that occur at different levels of genomics, such as omics of hazardous variants, which may directly affect disease etiology. Over the past 20 years, a significant number of GWASs have been conducted in the area of asthma, leading to the breakthrough of several candidate genes for asthma threat [5]. Most markedly, the “17Q12-21” locus, which has been simulated in numerous GWASs and harbors numeral probable biologic candidate genes, comprising *ORMDL3* and *GSDMB*, has been concerned in the development of asthma. However, genetic loci for asthma typically only account for a small portion of the illness risk, and genomics by itself does not reveal a considerable amount about the larger biological setting in which the detected variations and linked genes function [6]. The various processes involved in the onset and worsening of asthma disease have been successfully characterized by other omics-based techniques. To outline various asthma endotypes, such as T2 high vs T2 low endotypes, transcript-omics, the systematic, unbiased assessment of expression of RNA across the genome, has been applied [7].

## Review

Epidemiology of asthma

Asthma diagnoses are rising globally at a rate of 50% per 10 years. Asthma impacts an estimated 300 million individuals worldwide now, and that number might rise to 100 million by 2025 [8]. As with most other illnesses, asthma prevalence is greatest in industrialized nations such as the USA or the UK. Table 1 explains asthma prevalence in developed versus developing countries [8].

**Table 1:** Asthma prevalence in developed versus developing countries [8]

Country	Prevalence/1,000
Scotland	183
UK	152
New Zealand	150
Australia	146
Canada	140
USA	129
Brazil	113
Pakistan	109
Turkey	73
France	69
Japan	68
Thailand	63
Germany	62
Nigeria	53
Iran	52
Malaysia	47
Italy	43
India	23
Russia	21
China	20
Macau	6

Most persons who get asthma before the age of 30, and around half of those who do before the age of 10 have the disease. Girls are half as likely as boys to have asthma while they are younger, but after puberty, females are more likely to experience it. In comparison to rural regions, metropolitan areas have a greater incidence of asthma [9]. Children's asthma is exacerbated by poverty and malnutrition, which impairs lung health. Population data provide evidence that asthma is a heritable characteristic. First, there are significant racial and regional disparities in the prevalence of illness. For instance, many Western cultures have high rates of asthma prevalence upwards of 20%, whereas people in developing countries have significantly lower occurrence rates as low as 1% or even lesser. The fact that various people have vastly varied environmental conditions is suggestive of a genetic basis for asthma [10]. Second, children of asthmatic parents have a greater risk of developing the disease. Children with one afflicted parent have a recurrence risk of around 25%, but children with both affected parents have a recurrence risk of about 50%. Asthma is likely to strike a person if that individual has a genetically near cousin who also has the condition, according to twin studies [11]. For instance, the difference in the relapse risk of asthma among monozygotic and dizygotic twins emphasizes the importance of genomic risk factors for asthma. However, the condition is that monozygotic twins' chance for asthma is only about 75% rather than 100%, suggesting that environmental risk factors also play a significant role [12]. The fact that asthma is hereditary and that offspring’s of asthmatic parents have a much higher chance of acquiring the disease, as well as the higher likelihood of concurrence among monozygotic twins compared to dizygotic twins, has long been used as evidence that asthma is heritable (genetic). Estimates of heritability ranged from 55% to 74% for adults and up to 85% for children [13]. These populace-based studies suggest genomic risk factors for an increased likelihood of developing asthma. However, the heritability is polygenetic in nature, with several different genomic variations influencing the chance of developing a disease [14]. This section provides a review of the present information about the genes and loci that promote the development of asthma, with a particular emphasis on linkage, candidate genes, GWASs, ad-mixture analysis, and new high-output sequencing methods. While it is clear that a person's chance of developing asthma is influenced by their family history, other genetic and environmental variables may also have an impact on how asthma manifests phenotypically [15]. It is believed that a limited number of genes determine each person's baseline risk, which is thereafter affected by a different set of controlling genes as well as external circumstances. For instance, individuals with asthma that is early onset are more likely to have a hereditary history of the condition than those with late-onset asthma, indicating that genes may affect the disease's beginning age [16]. In addition, there is evidence that the development and intensity of asthma, as measured by the occurrence of symptoms, lung function, level of airway responsiveness, and airway inflammation, aggregate within families, indicating that someone with a clear family history of asthma is increasingly likely to develop the condition themselves [17].

Pathophysiology of asthma

The lungs are the organ system that are impacted when someone has asthma. The lobes and segments that make up the lungs may be counted on one hand. The right lung contains 10 segments, whereas the left lung can have either eight or nine segments, varying on how the lobe is divided. Anatomically speaking, the respiratory is broadly classified into two areas: the conducting zone and the respiratory zone [18]. These zones are distinguished by their respective names. The conducting zone starts at the nose and continues all the way down to the bronchioles, while the respiratory zone begins at the alveolar duct and continues all the way down to the alveoli [19]. This is where gas exchange occurs. The bronchial tree is the principal organ impacted by asthma. The bronchial tree's primary purpose is to move air all through the lungs until it goes to the alveolar sacs. Asthma affects this process. The terminal end of the trachea gives rise to the bronchi, which then branch off into the left and right bronchi [20]. The diameter of the right bronchus is larger and oriented in a more vertical plane, while the left bronchus is more horizontal and has a lower diameter. The primary and primary branches of the bronchi subsequently branch off into secondary and secondary bronchi. In order to keep their walls intact, the bronchi include smooth muscle as well as elastic fibers, both of which are capable of contracting and relaxing in response to inflammatory mediators, bronchoconstrictors, and bronchodilators. This allows the bronchi to retain their structural integrity. As one moves further into the lungs, from the bronchi to the alveoli, the involvement of smooth muscle fibers increases significantly [21]. Lung elastance refers to the capacity of the lungs to revert to their resting state after being expanded, while lung compliance refers to the readiness of the lungs to expand when the respiratory system is functioning normally [22]. In people suffering from asthma, the inflammatory process causes a change in the physiologic mechanism, which results in a smaller radius of the airway. When all of these different systems operate together, they affect the compliance of the lungs in a way that makes it more difficult to breathe [23].

Molecular genetics of asthma

Asthma is linked to more than a hundred genes, while the number is continually expanding. The three primary categories of asthma susceptibility genes include function of the immune system, function of the mucosa, lung function, and progression of disease. If a gene has been linked to asthma in a research, it does not imply that the gene and the condition are causally related [24]. See Table 2 for estimation of heritability and loci for various diseases [25].

**Table 2:** Estimation of heritability and loci for various diseases [25] HDL, high-density lipoprotein

Disease	Number of loci	Proportion of heritability (%)	Heritability
Crohn’s disease	6	49	Sibling reappearance risk
Age-related macular degeneration	31	19	Genetic liability
Fasting glucose	7	14	Sibling repetition risk
Type 2 diabetes	19	7	Sibling repetition risk
Height	6	5.21	Residue phenotype
HDL cholesterol	41	4	Phenotypic variation
Early onset myocardial infarction	8	2.89	Phenotypic variation
Systemic lupus erythematosus	5	1.51	Phenotypic variation

ADAM33

*ADAM33* is the earliest positionally cloned asthma susceptibility gene, which means that its exact arrangement in the genome was established before the gene’s purpose could be established. Lung fibroblasts as well as bronchial smooth muscle cells both express *ADAM33*, a gene that is positioned on chromosome 20p13 [25]. When it was discovered for the first time in 2002, it was shown to be associated with both asthma and bronchial hyperresponsiveness. In recent years, its relevance has grown to include increasingly subtle aspects of the etiology of asthma, such as airway remodelling, the development of the illness, and with chronic obstructive pulmonary disease (COPD) [26].

Filaggrin

Filaggrin, a protein, helps keep the skin barrier robust. Filaggrin mutations have also been connected to the manifestation of allergic sensitization, hay fever, and asthma exclusively in those with atopic dermatitis [27]. Patients with atopic dermatitis have skin that absorbs allergens, which is necessary for the emergence of other allergic disorders including hay fever and asthma. It is noteworthy to mention that research has shown that skin barrier failure causes systemic allergy reactions, such as elevated IgE levels and hyperresponsive airways, in addition to increasing allergen sensitization [28].

Asthma Linkage

Family-based linkage and subsequently candidate gene organization analysis were initial methodologies of identifying disease vulnerability genes related to asthma. In the very first linkage research on asthma, the IgE responses causing asthma and atopy were linked to chromosome 11q [29]. Linkage studies often show a wider locus that encodes a number of putative genes, allowing one to concentrate on a particular gene of interest. Then, utilizing positional cloning, this bigger region is examined in further detail. Up till 2006, linkage and positional cloning studies identified eight important genes: *ADAM33*, *DPP10*, *PHF11*, *NPSR1*, *HLA-G*, *CYFIP1*, and *OPN3*. Linkage analysis, however, has weak power to unearth threat variants with a small effective size and mostly employs trios data, which limits the accessibility of additional specimens [30].

Asthma GWASs

The primary benefit of GWASs is their capability to carefully and objectively look into novel variants connected to a disease. GWASs, which have since 2007 found hundreds of genetic variants that may cause asthma, have enabled the creation of a systematic GWAS of disease vulnerability variations [31]. The very first GWAS on asthma that was carried out included 900 patients with asthma that started in infancy and 1,245 healthy controls with European ancestry. After genotyping approximately 317,000 SNPs, a novel risk locus on chromosome 17q12-21 was found [32]. The most frequently replicated asthma loci to far, 17q12-21, encodes a variety of genes, including *ORMDL-3*, *GSDMB*, *ZBPB2*, and *IKZF2*. These genes were indeed linked to asthma in subsequent GWASs and eQTL studies [33]. The vicinity of the locus has been widened to include surrounding regions that come under the potential asthma-related genes PGAP3, ERBB2, and GSDMA [34]. See Table 3 for potential asthma-related genes linked with ethnicities [34].

**Table 3:** Potential asthma-related genes linked with ethnicities [34]

No.	Gene	Ethnicities
1	ORMDL3	Caucasian
2	CTNNA3	Korean
3	IL1RL1	Caucasian/Korean/Taiwan
4	PDE4D	Caucasian
5	TLE4	Mexican
6	DENND1B	Caucasian
7	RAD50-IL13	Caucasian
8	IL1RL1/IL18R1	Caucasian
	ORMDL3	

Analysis of Transcriptomics in Asthma

As part of the transcriptome research of asthma, the expression of all RNA transcript types, mRNA, ncRNA, miRNA, in a certain cell or tissue type is profiled. The two primary techniques for doing transcriptomic research are RNA sequencing and DNA microarrays (RNA-Seq) [35]. The bulk of asthma transcriptome studies have been performed using DNA microarrays. Even though RNA-Seq is a cutting-edge equipment with a favorable future, its price is higher than that of microarray. The bulk of the sample categories are made up of asthma patients and healthy controls, and asthma cases can also be divided into subcategories such as mild, moderate, and severe [36]. The reproducibility of the results and the capacity to uncover marginal signals may both be improved by meta-analyses of several independent studies. Gene set enriched and network analysis may be used in the genes with differential expression (DEGs) to detect the pathways and gene networks that they are highly prone to influence [37].

Asthma Blood Cell Transcriptomics

Compared to more useful specimens from airways, blood cells may be readily accessible and obtained using less invasive techniques. Despite this, it has been revealed that blood cells express 80% of the genes found in the human genome and may act as sentries for diseases [38]. Another argument in favor of using blood cells is the advantage of them as a substitute for airway cells in the transcriptome profiling of asthma. The differently stated genes on bronchial epithelial cells and fibroblasts were likewise deregulated in peripheral blood mononuclear cells (PBMCs), according to a recent transcriptome research on severe asthma [39]. A differential gene expression analysis of WBCs from therapy-resistant chronic asthma, acute asthma, and healthy samples revealed that TAS2R pathways were considerably elevated in serious asthma. The whole-genome transcriptome analysis of asthma revealed thousands of DEGs; these DEGs are frequently examined using path and network studies to identify the useful operations of the genes impacting the pathophysiology of asthma [40]. The condition of asthma transcriptomic research across several tissue classes is outlined in the subsections that follow, along with a synopsis of some of the many discoveries in complex tissues.

Asthma-Related Airway Epithelial Cell Transcriptomics

Airway cells, which also perform a direct role in asthma manifestation, are principally responsible for the onset of inflammation, hyperresponsive, and remodelling abnormalities in asthma manifestation [41]. Numerous transcriptome analyses of asthma had been performed on airway cells from various divisions. Through cluster analysis of the DEGs, two substantial gene signatures linked with aggravations were identified [42]. One cluster was characterized by gene signatures linked to innate immunity pathways, and the other cluster was described by gene signatures associated with lymphocyte initiation via antigen receptors and successive downstream results of adaptive immunity [43]. Although the effect was greater in the latter group, severe and mild or moderate asthmatics had comparable and extremely related differential expression patterns in blood cells [44].

Asthma Sputum Transcriptomics

Transcriptome study of the sputum from asthma patients with neutrophilic airway inflammation indicated enhanced manifestation of various genes linked to TNF signalling [45]. Neutrophilic asthma exhibited basic TNFR1 and TNFR2 levels that were significantly greater than non-neutrophilic asthma. The study also discovered that enhanced sputum TNFR1 and TNFR2 were linked to decreased lung function and asthma control in persons with severe asthma, while sputum and serum TNFR2 were linked to more frequent exacerbations [46]. The higher sputum-soluble TNF receptor levels in neutrophilic asthma suggest that airway monocytes might be playing a role in the deregulation of the TNF pathway, according to the transcriptome study of blood and sputum [47].

Asthma RNA Sequencing

RNA-Seq data have been used in a variety of different asthma transcriptome investigations, which have all been documented. RNA-Seq is a relatively new method being used in this field. On whole-genome transcriptomic analysis employing RNA-Seq technology on peripheral blood, PTGDR2 was shown to be a substantial biomarker of adult asthma [48]. This discovery was made by the researchers. The study found that there was a substantial up-regulation of PTGDR2 in the subsets of allergic asthma. During cold-related asthma aggravations in children, an RNA-Seq analysis using data from blood and nasal lavage was carried out as part of a potential, longitudinal case-control study. The purpose of this investigation was to discover changes in gene transcription [49]. Other enhanced pathways, including those linked to ferroptosis, adherens junctions, gap junctions, TGF-signalling, and the herpes simplex virus infection, were also found by the investigation [50].

Asthma Single-Cell RNA Sequencing

The capability to solve transcriptional profiles of the whole transcriptome right down to the level of the specific cell is the most intriguing advancements in gene expression research that has occurred to date [51]. The expression of certain transcripts may be utilized to differentiate between cell populations, and differences in disease states can be linked to variances in cell populations and their transcription [52]. Recent asthma research has begun to make benefit of this level of information. It was shown that numerous proinflammatory genes, such as *JAK1*, *NEAT1*, and *IL32*, were markedly expressed in CD8+ T cells, CD4+ T cells, NK cells, and B cells of severe asthmatics using scRNA-Seq followed by transcriptome analysis to profile PBMCs of severe asthmatics and controls [53]. This was demonstrated by comparing the PBMCs of severe asthmatics and controls. The transcriptome profile showed a degree of heterogeneity that was also specific to the individual cell types. For example, people with severe asthma had higher levels of CMKLR1 in their natural killer cells, but their monocytes had lower levels of this receptor [54].

## Conclusions

Asthma is one of the most dangerous and mysterious allergic disorder. Asthma is a complicated multifactorial illness with hereditary and environmental influences that tend to cluster within families. Parental cognation, serum intracellular cell adhesion molecule-1, and *ADAM33* were considerably linked with asthma, although the ABO blood system, IL-4, and serum E-selectin were not. The initial pedigree analysis showed that autosomal recessive pattern of inheritance was noticeable in asthma. Asthma has been linked to more than a hundred loci, and there are hints that a main gene might get mutated to cause asthma. Multi-ethnic cohorts along with deep phenotyping, longitudinal phenotypes, improved categorization of environmental factors, as well as the use of newer technologies and analytic approaches in the integration of "omics" data, will be required in the future for improved asthma prediction, diagnosis, and biomarker development. Next-generation sequencing techniques, such as DNA-Seq and RNA-Seq (including scRNA-Seq), better bioinformatic tools, and epigenomic procedures in the appropriate tissues/cells are some examples of these. Therefore, the clinical complexity and the etiology of asthma may be resolved through the methodical incorporation of omics data (for example, genomic) using multi-ethnic patient data from providers (for example, electronic medical records) and non-providers (such as monitoring tools that trigger when there is a change in the environment). The term "precision medicine" refers to a medical practice that goes well beyond the analysis of genetic sequences. When multi-omics data are combined with detailed phenotyping and the clinical results of cohort studies, this opens the door to a greater framework of methods and techniques in conditions such as asthma that are difficult to treat.
